# Buprenorphine Microdosing Cross Tapers: A Time for Change

**DOI:** 10.3390/ijerph192416436

**Published:** 2022-12-08

**Authors:** Amer Raheemullah, Ori-Michael Benhamou, Jamie Kuo, Anna Lembke

**Affiliations:** Department of Psychiatry and Behavioral Sciences, School of Medicine, Stanford University, Stanford, CA 94305, USA

**Keywords:** chronic pain, cross taper, microdose, microinduction, buprenorphine, induction, opioid use disorder, addiction, overdose, opioid

## Abstract

Buprenorphine is a partial opioid agonist that is Food and Drug Administration (FDA) approved to treat chronic pain and opioid use disorder (OUD). The national prescribing guidelines in the United States (US) recommend that patients transitioning from full opioid agonists to buprenorphine first undergo 12 or more hours of active opioid withdrawal, in order to avoid buprenorphine-precipitated opioid withdrawal. This opioid-free period imposes a significant barrier for many patients. Evidence is accumulating that using microdoses of buprenorphine to cross taper from full-agonist opioids to buprenorphine is a safe and effective way to avoid opioid withdrawal and uncontrolled pain. This microdose cross-tapering strategy is already being used across the US. The US prescribing guidelines and buprenorphine training would benefit from acknowledging this new approach. Additionally, to facilitate this strategy, the FDA should approve transdermal buprenorphine formulations for OUD and manufacturers could produce lower dose formulations of sublingual buprenorphine. The time has come for us to embrace buprenorphine microdosing cross tapers as a new standard of care.

Buprenorphine is a partial opioid agonist that is Food and Drug Administration (FDA) approved to treat chronic pain and opioid use disorder (OUD). It has become an important tool in the nationwide effort to combat opioid addiction and death from an overdose. The national prescribing guidelines in the United States (US) typically recommend that patients transitioning from full opioid agonists to buprenorphine first undergo 12 or more hours of active opioid withdrawal, in order to avoid buprenorphine-precipitated opioid withdrawal.

Requiring patients to enter into opioid withdrawal before starting buprenorphine imposes a significant barrier for many patients. This barrier is magnified in patients dependent on opioids for pain control, and in patients with cardiovascular disease who cannot sustain the associated cardiac stress. Similarly, patients with significant psychiatric morbidity may not tolerate the dysphoria or anxiety of withdrawal. Yet, the effective treatment of these comorbidities is often not feasible until patients have been transitioned off of other opioids onto buprenorphine.

Evidence is accumulating that starting very low doses of buprenorphine to transition from full-agonist opioids to buprenorphine is a safe and effective way to cross taper onto buprenorphine and avoid opioid withdrawal and uncontrolled pain. These very low doses of buprenorphine are often referred to as “microdoses” in the literature. This method was first described in 2010, followed by published case reports and case series, demonstrating the effectiveness and tolerability of using different dosing schedules, delivery methods (sublingual and transdermal), and settings (outpatient and inpatient) [1]. According to the most recent literature review, this microdose cross-tapering strategy does not precipitate rebound pain and is already being used across the US, including at many top academic institutions, for patients with chronic pain [1].

We believe national guidelines in the US should embrace this new buprenorphine cross-tapering approach and provide basic guidelines for how and when to use it. To facilitate the proper use of this approach, buprenorphine training programs should be updated, transdermal formulations for OUD should be approved by the FDA, insurance coverage should be broadened to include microdosing formulations, and very low dose sublingual formulations (less than 1 mg) should be made commercially available in the US.

Specialized training is mandated by the US Drug Addiction Treatment Act of 2000, in order to obtain permission to prescribe buprenorphine for OUD. Current buprenorphine training programs describe precipitated withdrawal as an inevitable consequence of buprenorphine’s partial agonism and higher binding affinity to the µ-opioid receptor. In fact, continuing a patient’s other opioids, starting with a small enough dose of buprenorphine, and cross-tapering slowly, can bypass precipitated withdrawal. Small doses of sublingual buprenorphine, typically 0.5 mg or less (but even up to 1 mg), do not precipitate withdrawal [1]. One study measured the vital signs and validated withdrawal scales in subjects who were receiving methadone concurrently with 0.2 mg of intravenous buprenorphine [2], and there was no difference in these subjects compared to the placebo group. Updated training should acknowledge this important relationship between the starting dose and precipitated withdrawal and highlight the option to use buprenorphine microdose cross tapers. It should help distinguish when a microdose cross-taper is more appropriate (e.g., chronic pain patients who cannot tolerate a painful opioid-free period) and when a traditional induction is more appropriate (e.g., patients already in enough opioid withdrawal to start larger doses of buprenorphine directly).

Cross tapers are commonplace in opioid management in general and are also safe with buprenorphine in particular [1,2,3,4,5]. Buprenorphine cross tapers differ in that they start with titrating up very low doses of buprenorphine (to avoid precipitated withdrawal), while full-agonist opioids are maintained at the same dose for some time, and only titrated down after buprenorphine has reached an adequate dose [1]. However, they are similar to any other opioid cross taper in that the cross-taper plan will be different for each patient’s unique clinical circumstances, such as the patient’s baseline opioid formulation and dose, the clinical setting, comorbidities, and the presence of OUD or chronic pain. Studies may continue to delineate the optimal delivery method and speed of the titration of buprenorphine cross tapers in special populations and clinical settings. However, we argue that the existing evidence and expert consensus are currently sufficient to support a microdose cross-taper without an opioid-free period [1,2,4,5]. Especially when many patients who need buprenorphine may decline a traditional induction because it is too painful but agree to a microdose cross taper [3].

Buprenorphine microdose cross tapers are being conducted most commonly with sublingual and transdermal formulations, but intravenous and buccal formulations have been used as well [1]. Sublingual formulations are used most often in microdose cross tapers and are generally more cost effective than transdermal patches in the US [1]. Since the lowest commercially available sublingual dose can precipitate withdrawal, the available formulations are cut by patients into smaller partial doses that do not precipitate withdrawal [4,5]. The most common initial partial sublingual dose used is 0.5 mg (1/4th tablet/film) [1]. However, cutting the pills and/or films lacks precision and can generate uncertainty and confusion during the cross taper, and high-performance liquid chromatography analysis only showed a content uniformity in films cut in half [1,6]. Smaller doses and microdosing buprenorphine starter packs could be made commercially available to support buprenorphine cross tapers without the need for an opioid withdrawal [4]. Starter packs and escalating dose strategies are routine in addiction treatment, as seen with varenicline and bupropion for nicotine use disorder and naltrexone for alcohol use disorder.

Transdermal buprenorphine is the next most commonly used formulation and some institution’s pharmacies prefer it because they do not allow for the administration of partial doses of sublingual formulations [1]. Transdermal buprenorphine delivers small hourly doses that do not precipitate withdrawal in opioid-dependent patients [3,5]. However, transdermal formulations are FDA approved for pain, not opioid use disorder. The FDA should broaden the usage label of transdermal buprenorphine to include OUD, to be used as part of the induction phase.

## Conclusions

The prescribing practices for buprenorphine in the US have been evolving as we learn more about the drug’s properties. For example, although buprenorphine started as a treatment for pain, it evolved with the evidence into a treatment for OUD. The initial prescribing guidelines required the in-office supervision of a buprenorphine induction, then evolved to permit home-inductions. Patient-per-prescriber limits have increased over time from 30 patients per prescriber to 275 for those with advanced training.

The time has come to re-examine once again our practices and attitudes. Opioid withdrawal as a necessary part of transitioning onto buprenorphine may be a remnant of a “War on Drugs”, when drug addiction was still seen as a moral failing requiring atonement through punishment, rather than a disease state. Buprenorphine microdose cross tapers are the de facto practice across the United States [1]. It is time for prescribing guidelines to embrace this strategy as a standard of care for all.

## Data Availability

Not applicable.
